# Rubber Dam—Its Use and Application

**Published:** 1873-01

**Authors:** L. G. Noel


### Dental Register.

VOL. XXVII.] JANUARY, 1873. No. 1.]

### COMMUNICATIONS.

### Rubber Dam—Its Use and Application.

BY L. G. NOEL, M. D., D. D. S.

*Read Before the Tennessee State Dental Society.*

In volunteering an essay on the Use and Application of Rubber Dam, I have not chosen a mean or narrow subject, but one that is worthy of the profound attention of all who are striving to attain higher degrees of perfection in dental operations. I think I may safely say that it is, at this time, claiming the attention of the greater part of the profession, and is being used by all who are making any pretensions to contour and difficult proximate fillings. It would seem a work of supererogation to enter into an argument to prove the usefulness of this great dessicator of carious cavities; yet there are dentists scattered all over this country, who have fought the oral fluids with spunk, napkins, and bibulous paper since the beginning of professional life, who have sadly elt the need of something more perfect than any of the thousand appliances introduced to meet this end, and yet who turn away from the only *perfect* appliance that has ever been invented, and pronounce it worthless. They say it is difficult of application; trying on the patient; that too much time is consumed in its adjustment; that its odor is objectionable; and there is no end to the objections that are raised to it, by those who do not know its value. Some object that it is only applicable to certain cases; and others go so far as to say it is not perfect, and does not meet the requirements in any. That it is difficult to apply in many situations, and quite improbable in others, no one will deny; but are we to refuse to use it in those cases where it is a blessing, and a boon beyond price, because it is not universally applicable? Are we to lay it aside because our patients are worried sometimes in its adjustment? In excavating we would continue to cut until the cavity was properly shaped to retain the filling, regardless of the pain we give our patient; then why consult his comfort if the rubber is the only means of attaining a perfect result? Then this objection only obtains to a limited extent with those who have had experience in its use, for such skill is rapidly acquired as enables the operator to apply it with ease and facility in all ordinary cases. As the question of time, no *honest* dentist will permit this to weigh with him a moment. We owe it to our patients to provide ourselves with the best appliances and materials, and to use them to the best of our ability, regardless of time, labor, or expense. But one says, I use soft foil altogether, and therefore have no use for rubber. To him I would say, your error in using soft foil *altogether* is as great as that of neglecting the dam. I do not pretend to say that soft foil has had its day (a broad assertion made by Dr. C. R. Taft, in the March number of the DENTAL REGISTER), for I believe there are cavites that can be better filled with soft foil than with any other form of gold. But in all mesial and distal cavities, in the bicuspids and molars, in the majority of buccal and crown cavities, and in *all* cases of contour fillings, I am convinced that adhesive gold is preferable. Every man in this society, who makes any pretensions to operative dentistry, ought to know that better operations can be made with gold in all cases of compound cavities in the bicuspids and molars, that can be made of soft foil; this too without making those large V shaped separations, by which so much tooth structure is lost. Now what man in this society does not know that it would be sheer nonsense to talk of filling with adhesive gold a compound cavity, or any other giving promise of requiring any considerable length of time, without the rubber? This is more particularly true of the inferior teeth, where from the dependency of the parts the saliva will accumulate, and overflow every other barrier. I am aware that many of these teeth are being filled without the rubber, but I have no confidence in fillings that are crowded in such made haste as the operator must make, where he expects every minute to see the cavity overflow, and his work spoiled. I have heard of gold being welded in contact with saliva, but have observed that all who claim to be able to perform this feat use the dam where they can get it on; thus proving their want of confidence in such welding. I wish to make a few practical suggestions with regard to the application of the dam, and shall endeavor to give such ideas as in my limited experience, I have been able to pick up; trusting I may have gleaned something that will be of use to some one who is seeking light on the subject. The first point inviting attention is the selection of a piece of rubber of good quality, and of suitable size for the case. In regard to quality, I would remark, that the best article now in the market is liable to oxidize and deteriorate, unless kept tightly wrapped, free from exposure to air, and in a cool place. It looses its elasticity and toughness rapidly when exposed to the air. In the selection of a piece some judgment is necessary in regulating its size. For instance, you have occasion to apply it to a second inferior molar, the rubber must extend posteriorly a sufficient distance to preclude the possibility of the patient getting the tongue over its margin. For the superior teeth it need not be so large. Holes may be cut folding twice, so as to protrude a sharp corner, which may be clipped off with a pair of shears, or by plunging the small handle of an excavator through the rubber, which causes a small piece to fly out, leaving a round hole. This, however, is only applicable to cases where it is wanted over one tooth, owing to the difficulty of punching the holes at exactly the point desired. The best instrument for punching the holes is one made after the form of a conductor's punch. With this they can be made with well defined margins, and at any point desired. Where there is an uninterrupted arch it is better to put it on two or more teeth. This becomes absolutely necessary in cases of proximate cavities, for reasons that are obvious. If the teeth are widely separated at the necks, the holes must be made wide enough apart to afford a covering or rubber for the festoon of gum between; otherwise leaking will occur at this point. In applying the dam little difficulty is experienced in getting it on the tooth, but it is frequently difficult to retain it in position, particularly if they be a little cone-shaped, having the base of the cone for their necks. This difficulty is overcome by passing a piece of waxed floss silk, shoe thread, or gilling twine, between the teeth. This is the only way to work the dam down between them in many situations, and is always the best way to tuck it under at their necks, which is a point too often overlooked by many who are using it. Often the only way to retain the rubber in position is to apply a ligature to the necks of the teeth to which it is adjusted. It is surprising how so small a barrier as that offered by a slender thread of waxed silk will hold the elastic rubber at bay. In tying the ligature, the surgeon's knot, or clove hitch of the sailor, should be used, as it prevents the slipping of the first knot until the second is made. The ligatures answer the double purpose of holding the dam in position and of tucking under its margins. After tying the ligatures they may be pushed under the margin of the gum, with the end of a burnisher, carrying the edge of the dam before them. This effectually prevents any leakage; I apprehend that some who have failed with the rubber have overlooked this point. The ligatures give the operator free use of both hands, as there is no longer any necessity for holding the rubber on with the fingers of the left hand. Wedges driven in between the teeth after putting the rubber on, answer a good purpose in many instances, and in like manner pieces of spunk and bibulous paper may be tucked between them. They not only hold the dam in position, but take up any moisture that may from its imperfect adapation ooze through. There are cases in which I believe it is impossible to adjust the dam, but these are confined chiefly to the wisdom teeth. Their shape is generally very unfavorable to its retention, being in most cases larger at their necks than at their grinding surfaces. Then the absence of any interstice posterior to them, into which to pass the string, and the increased tension brought upon the rubber as we pass back to that region of the mouth, both tend to augment the difficulties of holding it in position. When the tooth is somewhat isolated from its fellow, a slip noose may be passed over it, and a weight suspended to it to keep up continued tension; or if it be an inferior tooth the string may be given to the patient to hold. This plan works admirably in many cases besides the one cited, to which the ingenuity of the operator may adapt it.

Another method of surmounting this difficulty is set forth by a correspondent in the June number of the *Cosmos*, and though I have not yet put it to actual test, I am inclined to think it is a good idea. His method is to stretch the rubber over a stick, or instrument handle, about the size of the tooth, and threading a needle with waxed floss silk, lace the hole all around, coming back to the starting point, when the needle is passed through a loop in the end of the thread. We now have a slip noose, interlaced with, and which may be put on with the rubber. It is now taken off the stick, and applied to the tooth, when by drawing upon the string the rubber is drawn up tightly around its neck. Sometimes we meet with proximate cavities, extending between the margin of the gum, to such an extent that it is difficult to expose their gingival margins. This is managed by dissecting away a portion of the gum before adjusting the rubber.

In conclusion, gentlemen, let me urge those of you who are not using this valuable acquisition to our armamentarium to take it up and try it, and my word for it, as you pass from the incisors and canines to the bicuspids and molars, you will acquire such skill in its application as will put a stop to the complaints of your patients, and will so increase your ability to benefit them, in your operations that they will call down blessings upon your head an l you upon the head of its inventor.

